# Chromatic Signals Control Proboscis Movements during Hovering Flight in the Hummingbird Hawkmoth *Macroglossum stellatarum*

**DOI:** 10.1371/journal.pone.0034629

**Published:** 2012-04-17

**Authors:** Joaquín Goyret, Almut Kelber

**Affiliations:** Lund Vision Group, Biology Department, Lund University, Lund, Skåne, Sweden; University of Sussex, United Kingdom

## Abstract

Most visual systems are more sensitive to luminance than to colour signals. Animals resolve finer spatial detail and temporal changes through achromatic signals than through chromatic ones. Probably, this explains that detection of small, distant, or moving objects is typically mediated through achromatic signals. *Macroglossum stellatarum* are fast flying nectarivorous hawkmoths that inspect flowers with their long proboscis while hovering. They can visually control this behaviour using floral markings known as nectar guides. Here, we investigate whether this is mediated by chromatic or achromatic cues. We evaluated proboscis placement, foraging efficiency, and inspection learning of naïve moths foraging on flower models with coloured markings that offered either chromatic, achromatic or both contrasts. Hummingbird hawkmoths could use either achromatic or chromatic signals to inspect models while hovering. We identified three, apparently independent, components controlling proboscis placement: After initial contact, 1) moths directed their probing towards the yellow colour irrespectively of luminance signals, suggesting a dominant role of chromatic signals; and 2) moths tended to probe mainly on the brighter areas of models that offered only achromatic signals. 3) During the establishment of the *first contact*, naïve moths showed a tendency to direct their proboscis towards the small floral marks independent of their colour or luminance. Moths learned to find nectar faster, but their foraging efficiency depended on the flower model they foraged on. Our results imply that *M. stellatarum* can perceive small patterns through colour vision. We discuss how the different informational contents of chromatic and luminance signals can be significant for the control of flower inspection, and visually guided behaviours in general.

## Introduction

Visual systems assess the configuration of the environment based on the detection of different *quantities* of light (brightness; achromatic vision), different *qualities* of light (colour; chromatic vision), or both. The topographic nature of image-forming vision and the spatial limitations for photoreceptor arrangement impose some constraints, particularly for chromatic vision. To assess the colour of a point, or “pixel”, in the visual field, it is necessary (though not sufficient) that incident light be detected by different photoreceptor types, sensitive to different, relatively narrow ranges of the electromagnetic spectrum (e.g. UV-, blue-, and green-sensitive photoreceptors in many insects). On the other hand, to assess the quantity of light, or brightness, from the same point, light can be gathered by one or more photoreceptors of a single type (usually with a relatively broader spectral sensitivity), whose excitation contributes to the same signal (green-receptor channel in insects studied so far). Therefore, chromatic vision generally is less sensitive and has lower resolution than achromatic vision, which results in constraints to detail detection and, some effects on temporal resolution [1]. Thus, tasks involving detection of small (or distant), or moving objects tend to be performed using achromatic contrast (in honeybee: [2], in chicken: [3], in goldfish: [4], in budgerigars: [5]).

Nevertheless, colour vision is widely spread, which suggests colour discrimination to be of great importance. Moreover, chromatic signals tend to generally be used in visual tasks involving object recognition. Thus, colour signals can be significant for mate-choice [6], hunting and aposematism [7], aggressive territorialism [8], and host detection [9], [10], [11].

This apparent “specialization” to different visual tasks of colour (object recognition) and luminance (movement and detail detection) signals is independently supported by evidence that in humans and insects the processing of chromatic and achromatic signals appears to be through different physiological pathways ([12], [13], reviewed in: [14], [15]).

Among insects, pollinators are relatively well studied in regards to the visual signals used to visit flowers, particularly hymenopterans such as honeybees [16]–[18] and bumblebees [19]–[21], but also Dipterans [22] and Lepidopterans [23]–[25]. Even so, the factors determining the use of achromatic cues in insects capable of colour vision are still elusive. Many visually guided behaviours demand high spatial and/or temporal resolution, and at these instances, achromatic vision seems to be more reliable [26], [27]. Nevertheless, some studies have shown that, for example, recognition of pattern orientation [28] in bees uses achromatic contrast even when spatial and temporal resolutions are far from their limits. Similarly, *Papilio* butterflies [29] and honeybees [30] use achromatic vision to land on flower models, even when they discriminate among them through chromatic cues.

Here, we evaluate the flower inspection behaviour of naïve free-flying *Macroglossum stellatarum* (hereafter: Macroglossum); a diurnal hawkmoth with trichromatic vision based on three photoreceptors with peak sensitivities in the same regions as most hymenopterans, i.e. UV, blue and green [31], [32]. Nectarivorous hawkmoths hover in front of flowers while they search for nectar with their long proboscis. At this instance, hawkmoths use visual input to control placement and movements of their proboscis on the flower, with floral markings, known as nectar or floral guides, having a strong effect in the behaviour [33], [34].

While the nocturnal hawkmoths *Manduca sexta* appear to assess these floral markings through an achromatic mechanism and strongly rely on mechanosensory cues [33], [35], the diurnal Macroglossum weight visual cues over tactile input. Moreover, it has been suggested that they could use chromatic cues during this behaviour [34]. This would be favoured by the high luminance conditions during their active periods and the relatively short distance to the flower at which they forage (proboscis of ∼2.5 cm). Nevertheless, it would challenge our notion that visual stimuli used in motion detection and self-motion control, and form/pattern perception, are typically governed by achromatic contrast (reviewed in: [14], [36]).

We presented Macroglossum with flower models that had coloured patterns offering chromatic and/or achromatic contrast to investigate which of these visual cues might be of relevance for proboscis placement and its subsequent movements. By interchanging colour/brightness of the patterns and their corolla background we also evaluated the combined effects of colour/luminance signals and their relative position on the corolla. Additionally, we evaluated how availability of chromatic and/or achromatic contrast affected the efficiency with which moths inspected flowers, and their learning abilities for this task.

## Methods

### Animals

Larvae of *Macroglossum stellatarum* from our colony at Lund University were reared under a light:dark cycle of 16:8 hours on a natural host plant, *Galium mollugo*. We starved adults for 1–2 days in order to increase feeding motivation.

### Flower models

Circular flower models with a diameter of 3 cm were made out of paper (Ilford Galerie photo paper) and coloured using a Canon Pro9000 inkjet colour printer. At the centre of each flower we put a plastic “nectar tube” 2 cm long with an opening of 0.2 cm in diameter.

**Figure 1:**
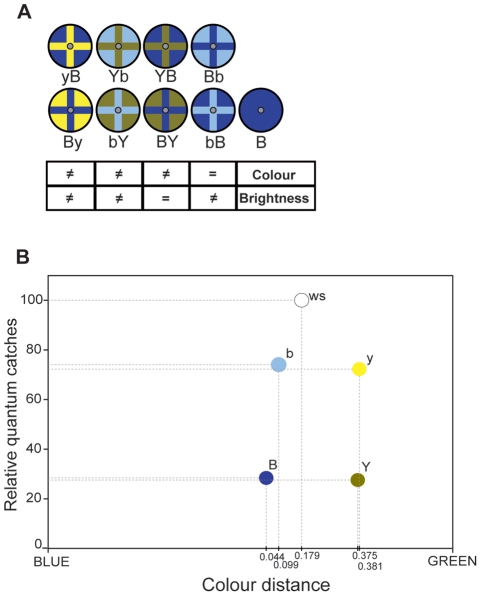
Flower models used in the experiments. A) Each model is named with 2 letters, where the first letter refers to the colour of cross mark and the second letter refers to the colour of the background “corolla” (B: dark blue; Y: dark yellow; b: bright blue; y: bright yellow). B) Relative catches (photon catches relative to a white standard -ws-) versus Colour distance (in the perceptual colour space of a hawkmoth) for the 4 colours used in flower models. See methods section for calculations.

We used 4 colours to produce the “corolla” of the different flower models, which were otherwise identical (Figs. 1A, B). These were “dark blue” (B), “bright blue” (b), “dark yellow” (Y) and “bright yellow” (y). We used blue and yellow because these colours have been shown to be (in this order) the preferred floral colours for hummingbird moths [25]. Spectral radiance (μW/steradian cm^2^ nm) was measured for each of these colours (range: 300–700 nm; intervals: 1 nm) with a spectroradiometer (International Light, RPS900-R) inside the experimental arena at a 45° angle and 5 cm apart from the flower model surface. We calculated the number of photons captured by each of the 3 photoreceptors in the retina of *M. stellatarum* (known as ultraviolet, blue, and green receptors for their peak sensitivity in the spectrum; Kelber and Henique, 1999) using the spectral sensitivity of *Deilephila elpenor*, a closely related hawkmoth, with peak absorption wavelengths: 350 nm (UV), 440 nm (Blue), and 525 nm (Green) [37]. This allowed us to determine the colour locus for each stimulus in the Blue-Green axis of the colour space (UV component was negligible due to the low UV emission of the light source; Fig. 1B), and the perceptual “distance” between colours (non-dimensional). We used the calculated number of photons captured by the green receptors to assess the achromatic contrast between stimuli (Fig. 1B;[1]). Achromatic contrast (C) was calculated as: C_1/2_ = (Q_1_−Q_2_)/(Q_1_+Q_2_); where Q_1_ and Q_2_ are the quantum catches of green receptors for colour 1 and colour 2, respectively. As shown in figure 1B, dark blue (B) and dark yellow (Y), as well as bright blue (b) and bright yellow (y), offer practically no achromatic contrast (C_B/Y_ = C_b/y_ = 0.01). On the other hand, bright blue (b) and dark blue (B) have basically no chromatic contrast between them, with colour loci 0.055 units apart. Similarly, bright yellow (y) and dark yellow (Y) are only 0.006 units apart, offering practically no chromatic contrast.

With these colours we produced 8 flower models of which 7 were bicolour. One colour was used as the “floral marking” in the shape of a cross (arms width: 0.5 cm) and the other as the “corolla background” (Fig. 1A). The models were: bright yellow cross on dark blue (yB – nomenclature: first character for the cross and second character for the corolla background), dark yellow cross on bright blue (Yb), bright blue cross on dark yellow (bY), dark yellow cross on dark blue (YB), dark blue cross on dark yellow (BY), dark blue cross on bright blue (Bb), bright blue cross on dark blue (bB), and plain blue (B). Additionally, we include the dark blue cross on bright yellow (By) used in a previous, methodologically identical study for comparison [34].

The original experimental design included models combining bright yellow with dark yellow (only achromatic contrast) and bright yellow with bright blue (only chromatic contrast), but naïve moths were very unresponsive to these colour combinations. Because here we evaluated innate behavior, we were compelled to use only the models to which naïve moths responded readily. We used a cross as the floral pattern because in animals that show a preference for one of the 2 colour/intensities presented, this pattern is effective at revealing how inspection efficiency can be affected by sensory biases [33], [34]. Additionally, the cross pattern is also comparable with common radial patterns seen in nature, and are very attractive to hummingbird hawkmoths [38]. This resulted in an appropriate experimental design to test our hypotheses.

### General procedure

The experimental arena consisted of a flight cage (height×depth×width: 65×65×80 cm) illuminated from above with fluorescent tubes (OSRAM Lumilux 18 W/965; see [39] for spectral distribution of illumination) giving an illuminance of 4280 lx (at the level of the flower models). In this arena we placed an array of 12 identical flower models, each with its corolla in a horizontal orientation 5 cm above the top of a green rectangular cardboard box (height×depth×width: 10×20×30 cm). Each model was filled with 5 μl of a 15% (w/w) sucrose solution. The walls of the arena were made of white cheesecloth, and the floor was covered with newspaper. One moth at a time was let to fly freely for 60 seconds. If it did not respond to the models (i.e. start probing) during this period, we presented it with a blue cardboard piece (2×2 cm; henceforth: primer) with a drop of sugar solution on its surface. Macroglossum are very responsive to dark blue objects, and after feeding, they become more responsive to objects of other colours, which otherwise do not elicit prompt responses. This procedure was performed to evaluate and increase foraging motivation [25]. If a moth did not respond to the primer, we captured it and did not include it in our analysis. If it responded, we let it feed for 2 seconds on it. If a primed moth did not respond to the models within 60 s, we captured it and recorded it as not responsive. If within that period a moth probed for more than 5 seconds, we recorded it as responsive, and let it forage for 180 more seconds.

### Statistical analysis

Responsiveness (as the percentage of moths that responded to our flower models) was tested by means of G-tests. Latency (as the time elapsed from take off until first probing event) was tested using one-way ANOVAs. To test empty flowers (as the number of flower models emptied during the foraging bout) we performed the non-parametric Kruskal-Wallis and Mann-Whitney tests because assumptions of the parametric models could not be met. Place of first touch with the proboscis was tested with binomial tests under the null hypothesis of no colour bias with P(cross area) = 0.41 and P(background corolla) = 0.59 (i.e. proportionally to their respective areas). Learning was evaluated by testing if probing time declined as the moths successfully inspected successive flowers (from the 1^st^ to the 10^th^) with a test of goodness of fit to an exponential decline function. Alpha-level for comparisons was corrected when multiple tests were performed (specified in Results for each case).

## Results

We tested a total of 193 moths, out of which 160 (82.9%) showed sustained flower inspection behaviours. There were no statistical differences in the responsiveness, latency or foraging time when comparing the different flower models (Table 1).

**Table 1:** Values and statistics for Responsiveness, Latency (mean±s.e.m.), Foraging time (mean±s.e.m.), Empty flowers (mean±s.e.m.).

	≠ Colour ≠Brightness			≠Colour = Brightness		= Colour ≠Brightness		
	yB	Yb	bY	YB	BY	Bb	bB	B
Responsiveness (%)	87.0	69.0	77.8	88.9	89.0	83.3	87.0	82.6
Latency time (s)	22±8	65±1	56±13	40±1	52±12	56±9	55±10	39±9
Foraging time (s)	180	180	180	180	180	180	180	180
Empty flowers	9.7±0.9^A^	10.3±0.8^Aα^	6.1±1.4^β^	11.2±2.6^A†^	9.7±0.8^†^	6.6±1.2^a^	10.1±1^b^	6.3±1.3^a^
Number of replicates (N)	23	29	18	25	27	24	23	24

Number of replicates indicates the number of moths that were exposed to each flower model (the base for the responsiveness percentages). The statistical tests are based on an α-level = 0.005 after a Bonferroni correction. Responsiveness (G-test): Gh = 5.52; p = 0.7; N = 193; Latency (ANOVA): F_(7, 155)_ = 2.64; p = 0.0135. Each comparison between models for the variable empty flowers is denoted by a superscript of a different type (A, a, α, and †). Statistically significant differences are denoted by different characters within each type (e.g. “a” and “b”, or “α” and “β”). Empty Flowers (Kruskal-Wallis tests): χ^2^ = 35.6; p<0.0001. Comparison among models with a yellow cross (A): χ^2^ = 2.35; p = 0.31. Comparisons among all models with only blue colour (a, b): χ^2^ = 10.79; p = 0.0045; only Bb vs. bB: χ2 = 10.13; p = 0.0015. Comparisons among “inverted patterns” Yb vs. bY (α, β): χ2 = 11.6; p = 0.0007; YB vs. BY(†): χ^2^ = 7.2; p = 0.0074.

### Chromatic and achromatic assessment of patterns by flower-naïve moths

**Figure 2:**
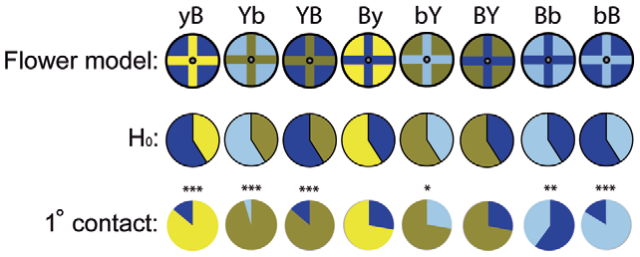
Area of first contact by flower-naïve moths. In the upper row are the different flower models; in the middle row are the expected distributions, for each model. These are colour-coded under the null hypothesis of no bias. In the lower row are the actual distributions based on the recorded data. *p<0.05; **p<0.01; ***p<0.00001. α-level  = 0.0063 after Bonferroni correction.

We evaluated the first contact on the flower surface of 223 moths. This includes moths that probed for less than 5 seconds (and thus, were not included in the analysis of the other variables; see Methods), and the *post hoc* analysis of previously recorded moths foraging on the By model [34]. In bicolour models naïve moths showed a very significant bias to first contact on the yellow cross independently of the achromatic contrast (Fig. 2; Binomial tests: yB, Yb, and YB: p<0.0001 in all 3 cases), as well as a strong tendency to first contact outside the blue cross (on the yellow background corolla) in the remaining bicolour models (Fig. 2; By: p = 0.18; bY: p = 0.03; BY = 0.07; Bonferroni-corrected α-level = 0.0063). These results show that these (and the previously observed [34]) biases for yellow are based on the chromatic assessment of the patterned flower models irrespective of achromatic contrast (see also *Learning and efficiency* subsection below). Nevertheless, the weaker tendency to first contact yellow as the background corolla than as a cross mark also suggests that *initial* probing of naïve moths tends to be aimed towards the floral marks, as shown by Lunau and collaborators in bumblebees and honeybees [40].

**Figure 3:**
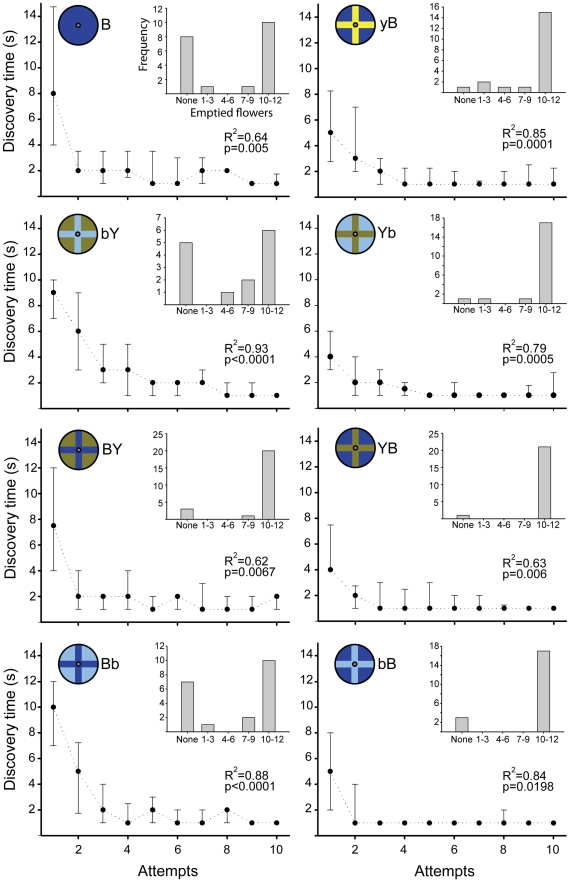
Discovery times (time elapsed (s) inspecting flowers, disregarding drinking and flying between models) vs. Attempts (1^st^, 2^nd^, …, 10^th^ successful nectary discovery events). R^2^ and p values are from tests for goodness of fit to an exponential decline function of 2 parameters (f(x) = a^(−bx)^), a typical learning curve. Insets: absolute frequency distributions of the number of successfully emptied flowers arranged in 5 bins (none, 1–3, 4–6, 7–9, and 10–12).

Moths could also assess floral markings through achromatic signals. On bB (no chromatic contrast) moths showed a very strong bias to first contact the brighter cross (binomial test: p<0.0001; fig. 2), while in Bb models they tended to first contact the dark blue cross (Fig. 2; binomial test; p = 0.0069; α-level = 0.0063). Interestingly, the initial bias towards the cross mark was stronger than in the bicolour models. Nevertheless, it is important to mention that in Bb models this initial bias was observed only at the *first contact(s)*. We directly observed moths probing mostly on the bright blue background of Bb models, which is also reflected in the different number of empty flowers for these models (compared with bB; Table 1 and Fig. 3). On bB, moths continued to probe on the bright blue cross, achieving a higher foraging efficiency (Table 1 and Fig. 3).

### Learning and efficiency

After finding 3–4 nectaries, moths learned to find subsequent nectaries in 2 seconds or less (Fig. 3). Nevertheless, while all models could be learned, the efficiency with which moths foraged on the different models was affected by the innate bias to probe on the yellow areas of bicolour models (frequency distributions in insets of fig. 3 and table 1).

In models with a yellow cross pattern on blue (yB, Yb and YB; Fig. 1A), moths found the first nectar tube relatively quickly (medians between 4 s and 5 s) and continued to decrease the inspection time thereafter (Fig. 3), resulting in high foraging efficiencies (see Table 1 for statistics). In models with a blue cross on yellow offering chromatic and achromatic contrast (By and bY), moths were initially slower, with median probing times until first success of 14 s and 9 s, respectively (Fig. 3; see [34] for data on By). The bias to probe on yellow also resulted in more moths finding fewer nectaries (bimodal frequency distributions in insets of Fig. 3). Thus, moths were less efficient overall, attaining significantly fewer nectaries when foraging in bY than in Yb (Table 1). Similarly, empty flowers was lower in By (mean±s.e.m. = 6.8±1.1) than in yB (mean±s.e.m. = 9.7±0.9; Table 1). These results suggest that the observed tendency to probe on the small cross mark regardless of its colour is relevant for the first(s) contacts only, and that subsequent inspection movements are primarily affected by colour differences. (We did not perform statistics for By vs. yB, because data from By was obtained from a similar, but previous experiment [34].) Moths spent more time to find the first nectary on BY (7.5 s) than on YB (4 s; Fig. 3), but the fast learning on BY accounted for a comparably high number of emptied flowers (Table 1). The fact that this occurred in the only pair with no achromatic contrast is suggestive, but whether luminance signals can interfere with learning or not has to be further investigated.

In bB (providing only achromatic contrast) moths found their first nectar tube relatively fast, and most animals could empty a high number of flowers (Table 1 and Fig. 3). On the other hand, when inspecting Bb flowers probing times until first successful event were twice as long than in bB (Fig. 3), fewer moths found nectaries (insets of Fig. 3) and, consequently, moths could empty fewer flowers than in bB (table 1). This suggests that after the first contact, moths directed their proboscis towards the brighter background areas, thus being “mislead from the nectar tube” by their innate achromatic control of inspection movements. This could also be directly observed during the experiments (personal observation).

## Discussion

Our experiments show that Macroglossum can use chromatic and/or achromatic signals to control the placement and movement of their proboscis during flower inspection. Our design allowed us to identify three, apparently independent, input signals affecting motor output. First, in bicolour blue-yellow models moths weighted chromatic over achromatic signals, probing on yellow areas regardless of their luminance contrast. This was directly observed, but also substantiated by the shorter probing times to find the first nectar tube (Fig. 3) and the higher foraging efficiencies on models with a yellow cross (intersecting the nectar tube) than in models with yellow as background colour (not intersecting the nectar tube; Table 1). Second, in blue models, providing only achromatic contrasts, moths probed more on the brighter areas, taking shorter times to find the first nectar tube, and achieving higher foraging efficiencies in models where the nectar tube was on the brighter cross (Table 1). Third, flower-naïve moths showed an innate bias towards the cross marks regardless of luminance or chromatic contrast during the establishment of the *first contact* with the corolla. This “spatial configuration” bias seemed to compete with (or enhance) the colour and luminance control in flower-naïve moths (Fig. 2). Nevertheless, it did not have a significant effect after the first contact, when probing showed to respond to colour or luminance biases for yellow and brighter areas, respectively. This is suggested by the higher foraging efficiencies (Table 1), and the lower inspection times needed to locate the first nectar tube (Fig. 3) when the cross marks were yellow (bicolour models) or brighter (in blue models). Thus, when considering only the first contact of flower-naïve moths on models with a yellow cross, the bias towards the mark seemed to enhance the bias for yellow (strong, significant bias toward yellow cross models; Fig. 2). Conversely, with a blue cross (bicolour models) both innate biases seemed to “compete”, resulting in weaker biases towards the yellow background (for first contact; Fig. 2) and longer inspection times until the first nectar tube was found (Fig. 3). For Bb models (only achromatic contrast) the scenario was analogous, but the bias to first probe the cross was stronger (compare Bb with bicolour models with a blue cross in Fig. 2). This suggests that the initial aiming at cross marks could be related to visual feedback mechanisms for flight control/stabilization using the achromatic channel, which, theoretically, is more reliable for tasks involving motion detection.

Nevertheless, during flower inspection the probing response of hovering moths was strongly affected by patterns whose edges and shape were only defined by chromatic signals. This was unexpected, because in insects studied so far, pattern, edge and form detection, as well as movement control, are handled through the more spatially and temporally resolving achromatic vision. Thus, our results strongly support the hypothesis that *Macroglossum stellatarum* can perceive small patterns through colour vision, as opposed to honeybees which do it through achromatic vision [13].

### What kind of information do chromatic and achromatic signals offer to a diurnal nectar forager?

Chromatic cues appear to be suitable for the assessment of object properties because they are relatively invariable under natural illumination conditions. This makes chromaticity a suitable ‘visual modality’ for object recognition and classification [34]. Luminance can vary greatly under changing illumination conditions, deeming it unreliable for these kinds of tasks [14], [36]. This seems to be reflected in the fact that insect pollinators usually have innate preferences for potential nectar sources that are based on chromaticity rather than on particular luminance levels. Also, both moths and bees have been shown to learn faster, and more reliably, colour than luminance [36].

Interestingly, luminance is, at least theoretically, a dimension of visual perception that can carry more information due to its greater dynamic range [16], [41]. Alternatively, the behavioural significance of this greater dynamic range could reside in the greater availability of luminance signals under different light conditions. In first place, the large illumination range under which achromatic stimuli can be used makes them reliable signals for control mechanisms, such as in flight stabilization, speed regulation and positional control. This becomes very relevant under low light conditions, or when fast motion is involved. Achromatic cues can be more widely used for object detection as in nocturnal pollination systems, where flowers offer high achromatic contrast with the vegetative background.

### Innate releasing functions of colours and motor output regulation

The strong innate bias towards yellow (independent of achromatic cues) is surprising, because Macroglossum have a robust preference for plain blue over plain yellow flowers [25], [32]. Consistently with the notion that spectral signals are more useful for object recognition, chromatic properties are typically associated with response ‘releaser’ functions, or “key stimuli” [42]. One hypothesis for this behaviour is that blue would act as an effective releaser of proboscis extension in flower-searching moths, while yellow would act as a releaser of the next behavioural stage, thus affecting proboscis placement and exploratory movements. As proposed earlier, this could be linked to the fact that ancestrally, anthophilous insects foraged on pollen (usually yellow) rather than nectar, which evolved later as a floral reward (see: [34]).

On the other hand, visual *contrast* could be the relevant signal. When hawkmoths approach and hover in front of a flower, their body position is regulated using visual contrast as feedback, such as looming stimuli [43]–[45], flower displacements [43], [45], [46], and optic flow from the background [47]. Contrasting floral markings could be offering a visual reference for the control of proboscis placement and movements in a similar way. In fact, parallel floral markings control the positioning of the hovering flight and proboscis in *Manduca sexta* hawkmoths [33]. Extensive research on motion, pattern, and edge detection suggests that insects use achromatic contrast feedback through the green receptor channel in regulatory motor responses (see reviews: [36], [48]). Our hypothesis implies a novel sensory-motor mechanism to control proboscis and/or hovering flight that involves chromatic contrast signals. We are now investigating this hypothesis by manipulating the degree of blend between marks and background (from sharp to graded boundaries), while recording with better temporal and spatial resolution.

### Achromatic signals

Moths were able also to use achromatic signals. In bB moths probed almost exclusively on the bright blue cross (Fig. 2), leading to more efficient foraging bouts (Table 1) and taking the shortest inspection times (Fig. 3). Conversely, first contacts on Bb were biased towards the dark blue cross, suggesting a substantial pattern-luminance effect on models with only achromatic contrast. This is consistent with the finding that Macroglossum prefers flowers with small marks regardless of whether they are the darker or the brighter than the background corolla as long as they offer high *luminance* contrast [49]. Nevertheless, after initial contact, moths mainly probed on the bright, background areas, which explains the poor performance on this model (Table 1).

### Flower inspection learning

Moths diminished the time spent inspecting on all of our flower models (Fig. 3). Nevertheless, the bimodality observed in some of the frequency distributions of the number of emptied flowers makes individual variation evident in some models (Fig. 3). In bicolour models where the nectar tube was placed in the “non preferred” colour (bY, BY), we observed 3 distinct behaviours. One group of moths would continue to probe chiefly on the preferred colour, avoiding the blue cross and thus, achieving low inspection success (larger numbers of moths that found none or few nectaries; insets of fig. 3). The other 2 groups changed their behaviour in 2 different ways, lowering inspection times and achieving higher efficiencies.

In one group, after a few successes moths started to probe on the blue cross and achieved higher inspection success. This is evocative of associative learning, in which animals associate a colour with the presence of a reward and modify their innate preferences. Presently, we cannot discard this hypothesis for this adaptive change of the inspection behaviour.

The last group, continued to probe on the innately preferred background (corolla) colour, but after finding the first nectaries, they started to place their proboscis equidistantly from the cross arms and dragged it in a straight line, towards the centre. This seemingly teleonomic movement appears to be using the markings as true guides, in the sense that their display was apparently used to orient both positioning and direction.

### What do hummingbird moths see when they inspect our flower models?

Macroglossum forages under daylight conditions, but its inspection behaviour involves extremely fast movements (preliminary recordings with a high speed camera show moths moving 25 mm in 100 ms while hovering over the contour of flower models and poking with their proboscis 150–200 times/s; supporting information Movie S1). The fast proboscis and body movements during inspection suggest *a priori* that moths would use achromatic visual feedback during inspection to control these motor outputs. This, in fact, *can* be the case; nevertheless, we have shown that Macroglossum can use small chromatic signals while hovering and probing on flowers.

One could interpret that contrast detection (chromatic and/or achromatic) alone suffices to explain results. If that were the case, colour inversions (e.g. bY-Yb) would not affect behaviour (and consequently, inspection efficiency) because inversions do not change the position, orientation, or length of contrast-lines. On the contrary, colour inversions showed that moths do not only assess contrast lines, but also the actual colour, shape, and relative position of the differently coloured areas. Naïve moths directed their proboscis and scanned within shapes (“central cross” or “outer triangles”), which were defined both by their chromatic contrast boundaries and their coloured inner areas.

Our study has uncovered complex interactions between colour, luminance, floral pattern, innate biases and learned inspection strategies. Nevertheless, we have shown evidence that hummingbird hawkmoths can detect floral patterns, and resolve their small shapes based only on chromatic signals. This allows them to precisely direct the fast exploratory movements of the proboscis and the body while inspecting flowers “on the wing”. Their ability to rapidly modify the use of visual signals adaptively, particularly chromatic ones (Fig. 3), reflects their switch to diurnal activity, and the generalist flower-visitation scheme that hummingbird hawkmoths carry on during their long dispersion from the Mediterranean to the northern latitudes during the boreal summer.

## Supporting Information

Movie S1 Slow-motion sequence of *Macroglossum stellatarum* probing on a flower model. The 11 seconds of playback (at a rate of 30 fps) correspond to 1.1 seconds in real time (recorded at a rate of 300 fps).
(MOV)
